# Comparison of Walking Protocols and Gait Assessment Systems for Machine Learning-Based Classification of Parkinson’s Disease

**DOI:** 10.3390/s19245363

**Published:** 2019-12-05

**Authors:** Rana Zia Ur Rehman, Silvia Del Din, Jian Qing Shi, Brook Galna, Sue Lord, Alison J. Yarnall, Yu Guan, Lynn Rochester

**Affiliations:** 1 Institute of Neuroscience/Institute for Ageing, Newcastle University, Newcastle Upon Tyne NE4 5PL, UK; rana.zia-ur-rehman@ncl.ac.uk (R.Z.U.R.); silvia.del-din@ncl.ac.uk (S.D.D.); brook.galna@newcastle.ac.uk (B.G.); sue.lord@aut.ac.nz (S.L.); alison.yarnall@ncl.ac.uk (A.J.Y.); 2 School of Mathematics, Statistics, and Physics, Newcastle University, Newcastle Upon Tyne NE1 7RU, UK; jian.shi@ncl.ac.uk; 3 School of Biomedical, Nutritional and Sport Sciences, Newcastle University, Newcastle Upon Tyne NE1 7RU, UK; 4 Department of Physiotherapy, Auckland University of Technology, Auckland 92006, New Zealand; 5 The Newcastle upon Tyne Hospitals NHS Foundation Trust, Newcastle Upon Tyne NE7 7DN, UK; 6 School of Computing, Newcastle University, Newcastle Upon Tyne NE4 5TG, UK; yu.guan@ncl.ac.uk

**Keywords:** Parkinson’s disease, machine learning, classification, wearables, accelerometer, GAITRite, multi-regression normalization, SVM, random forest classifier

## Abstract

Early diagnosis of Parkinson’s diseases (PD) is challenging; applying machine learning (ML) models to gait characteristics may support the classification process. Comparing performance of ML models used in various studies can be problematic due to different walking protocols and gait assessment systems. The objective of this study was to compare the impact of walking protocols and gait assessment systems on the performance of a support vector machine (SVM) and random forest (RF) for classification of PD. 93 PD and 103 controls performed two walking protocols at their normal pace: (i) four times along a 10 m walkway (intermittent walk-IW), (ii) walking for 2 minutes on a 25 m oval circuit (continuous walk-CW). 14 gait characteristics were extracted from two different systems (an instrumented walkway—GAITRite; and an accelerometer attached at the lower back—Axivity). SVM and RF were trained on normalized data (accounting for step velocity, gender, age and BMI) and evaluated using 10-fold cross validation with area under the curve (AUC). Overall performance was higher for both systems during CW compared to IW. SVM performed better than RF. With SVM, during CW Axivity significantly outperformed GAITRite (AUC: 87.83 ± 7.81% vs. 80.49 ± 9.85%); during IW systems performed similarly. These findings suggest that choice of testing protocol and sensing system may have a direct impact on ML PD classification results and highlight the need for standardization for wide scale implementation.

## 1. Introduction

Parkinson’s disease (PD) is a complex neurodegenerative disorder which progresses over time [1] and comprises both motor and non-motor symptoms [2], leading to poor disease management, poorer quality of life [3], and increased health care costs [4]. Early diagnosis of PD is critical for optimal management but remains challenging. Current diagnosis of PD is commonly based on subjective clinical examination (clinical scales) [5] often in conjunction with expensive and time consuming brain imaging techniques. Gait has been shown to act as a marker of global health and has been used to predict morbidity, mortality, falls risk and neurological disorders [6]. Recent work has shown that objective gait quantification of motor impairments can support PD diagnosis, also at an early stage [7,8].

Gait can be objectively quantified via a number of spatial-temporal characteristics and features [9,10,11,12]. In order to maximize information for disease classification, analysis of multiple characteristics can be enhanced using machine learning (ML) [6]. The most widely used ML models for PD classification are the support vector machine (SVM) and random forest (RF) [13,14,15,16,17,18,19]. Classification accuracy however is inconsistent across studies which may be largely due to methodological differences (e.g., testing protocols, gait assessment systems and normalization of participants’ data) [13,14,17]. This leads to difficulty comparing across studies and in turn to select the optimal gait protocol and outcomes for classification purposes. For example, protocols used to measure gait have different durations, distance and speed [10,20,21]. Moreover, gait assessment systems range from gold standards in the field of gait analysis using camera based motion capture and instrumented walkways [11,12] to wearable devices [22]. Practically, wearable sensors such as accelerometers, gyroscopes and magnetometers [23] have advantages as they are not context specific and can be used in the clinic and home [10,24]. This is relevant if gait proves useful for disease classification and clinical use.

For accurate disease classification the differences between participants should also be as low as possible and this requires normalization of the selected features as an important and critical step [16]. Between-participants gait differences are related to demographic characteristics such as the age [25], gender, and BMI [26,27]. Gait characteristics are also speed dependent [28,29,30] and normalization of gait features with respect to speed is usually performed [16]. Robust normalization processes thus optimize ML models and classification of PD [31].

The effects of walking protocol and gait assessment systems on the performance of ML models and the impacts on disease classification remain unanswered questions. The objective of this study was therefore to investigate the impact of different walking protocols and gait assessment systems on the performance of SVM and RF models for PD classification. We also highlight the strengths and limitations of protocols and devices to guide decision making in future studies. We compared the effect from two different walking protocols at normal pace (four times along a 10 m walkway (intermittent walk-IW); walking for 2 min on a 25 m oval circuit (continuous walk-CW)); and two different gait assessment systems: GAITRite vs. Axivity.

## 2. Methods

### 2.1. Participants

Data from the “Incidence of Cognitive Impairment in Cohorts with Longitudinal Evaluation - GAIT” (ICICLE-GAIT) study [11,32] encompassing 93 people with early PD and without dementia at study entry, and 103 healthy controls (HC) were included in this cross-sectional analysis. PD was diagnosed according to the UK Parkinson’s Disease Brain Bank criteria [21] by a movement disorder specialist [32]. The study was approved by the “Newcastle and North Tyneside Research Ethics Committee” (REC No. 09/H0906/82). All the participants gave their written informed consent before participating in the study. Experiments were conducted according to the declaration of Helsinki.

### 2.2. Demographic and Clinical Measures

Demographic characteristics such as age, height, weight, and BMI were recorded for all the participants. Cognition was assessed with the Mini-Mental State Examination (MMSE) [33] and balance confidence was evaluated with the balance self-confidence scale (Activities specific balance confidence scale; ABC) [34]. To assess PD motor severity, Hoehn & Yahr scale score [5] and the modified version of the Movement Disorder Society Unified Parkinson’s Disease Rating Scale (MDS-UPDRS)—section III [35] were used. Phenotypes in PD, namely postural instability and gait difficulty (PIGD), indeterminate (ID) and tremor dominant (TD) subtypes, were also calculated from MDS-UPDRS [36]. Levodopa equivalent daily dose (LEDD mg/day) was calculated according to defined criteria [37,38].

### 2.3. Walking Protocols and Data Collection

**Figure 1:**
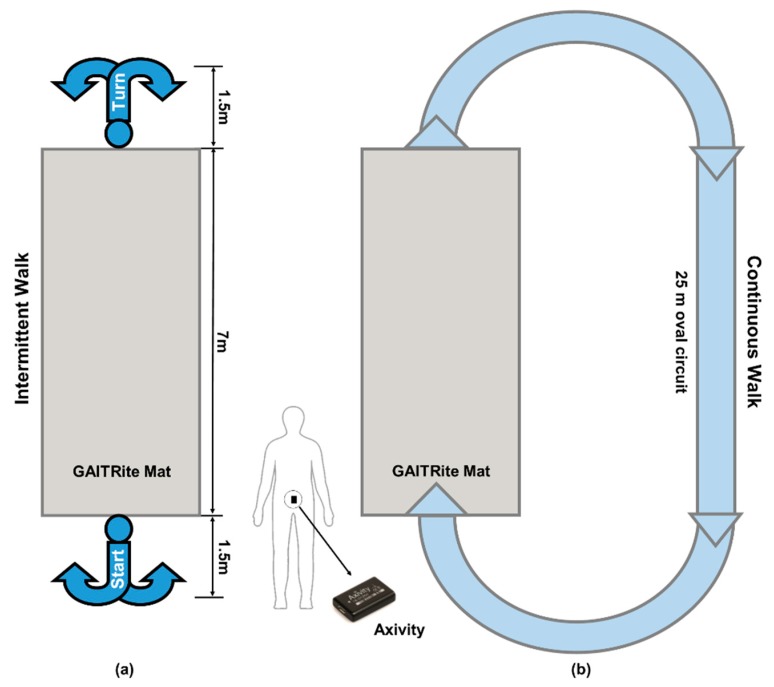
Layout of experimental setup and testing protocols, (**a**) 10 m intermittent walking test (IW); (**b**) 2 min continuous walking test (CW).

Two different protocols were used to assess gait. All PD participants were assessed one hour after medication intake:

(1) Ten meter (m) intermittent walking test (IW). Participants were instructed to walk in a straight line over a 10 m walkway (Figure 1a). They repeated this four times at their preferred speed. The GAITRite mat was placed in the center of the walkway [21].
(2) Two minute continuous walking test (CW). Participants were asked to walk continuously around at 25 m oval circuit at their preferred speed (Figure 1b).

### 2.4. Gait Assessment Systems

Each participant was asked to wear a tri-axial accelerometer (Axivity AX3, dimensions: 23.0 × 32.5 × 7.6 mm) on the lower back (L5), held in place with double sided tape (BSN Medical Limited, Hull, UK) [10]. The monitor measures the vertical, mediolateral and anteroposterior accelerations during walking at 100 Hz sampling frequency (±8 g range, resolution up to 13-bit). Data collected using Axivity were synchronized with real-time clock, and start and stop times of the trials were noted by the experimenter to automatically segment and analyze the accelerometer data via MATLAB^®^. Gait assessment was also conducted using an instrumented mat (Platinum model GAITRite: 7.0 × 0.6 m) [12]. GAITRite has a spatial accuracy of 1.27 cm and temporal accuracy of one sample (240 Hz, ~4.17 ms).

### 2.5. Data Processing and Gait Characteristics Extraction

From each testing protocol and gait assessment system, 14 gait characteristics were extracted [10,11]. Methods described in our previous work were used for extracting gait characteristics from the 10 m test and the 2 min test with GAITRite and Axivity [10]. For easy interpretation, these 14 gait characteristics were grouped into five domains (pace, rhythm, variability, asymmetry, and postural control) as described in our previous work [11].

### 2.6. Statistical Analysis, Gait Normalization and Classification Modeling

Multivariate analysis of variance (MANOVA) was performed on normalized gait characteristics to examine the main effect and interactions of group (PD vs. HC), walking protocols (IW vs. CW) and gait assessment systems (GAITRite vs. Axivity) on the gait characteristics. Independent *t*-tests were performed to understand the between-group (PD vs. HC) differences of demographic and gait characteristics to include as input to the ML model. Receiver operating characteristics (ROC) analysis was used to measure the discriminative power of each gait characteristic. Pearson’s correlation coefficients (r) were used to check the dependency among gait characteristics within each group. Distribution of each gait characteristic was plotted using rain cloud plots [39] for each group, walking protocol and gait assessment system. Gait characteristics were normalized for ML using multiple regression normalization [16] performed with respect to preferred gait speed (step velocity in each walking protocol from each gait assessment system), age, BMI and gender. This gave the ratio of the original and predicted gait characteristics based on the following equations:

(1) $$y_{i}=\;\beta_{0}+\beta_{1}*Gender_{i}+\beta_{2}*Age_{i}+\beta_{3}*BMI_{i}+\beta_{4}*StepVelocity{\left(speed\right)}_{i}+\epsilon_{i}$$

where $y_{i}$ is the gait characteristics from the 5 domains of the conceptual gait model, *i*th participant. $\beta_{0}$ is the intercept and β is the coefficient of the linear regression. $\epsilon_{i}\sim \;N\left(0,\sigma^{2}\right)$ is the residual for each participant *i*. For each testing task and sensing system, the model coefficients were estimated using the healthy control participants’ data based on Equation (1):

(2) $$y_{i}={\hat{y}}_{i}+\;{\hat{\epsilon }}_{i}$$

where ${\hat{y}}_{i}$ and ${\hat{\epsilon }}_{i}$ are the predicted value and residual error for the *ith* participant. Finally the normalized gait features were obtained by dividing the original independent gait feature with the predicted dependent gait feature by using following equation:

(3) $$y_{i}^{n}=\frac{y_{i}}{{\hat{y}}_{i}}$$

where $y_{i}^{n}$ is the final normalized gait characteristic for the *i*th participant and n is normalized. Based on the Taylor’s series the expected value of the control group normalized gait characteristics should be 1. The resulting gait characteristics will be unit less due to the division of $y_{i}$ and ${\hat{y}}_{i}$ as these have the same measuring units.

The support vector machine with radial basis function (SVM-RBF) and random forest were used because these are the most widely used ML models for PD classification [13,14,15,16,17,18,19,40]. The models were trained on the same conceptual features from both sensing systems to compare the impact of walking protocols and gait assessment systems. 10-fold cross validation repeated 100 times was used for the evaluation of the models. Single measure, area under the curve was used for the model evaluation [41]. Importance of the gait characteristics was identified by extracting the square of the weight of the gait characteristics in the SVM-linear classifier [42,43]. Gait characteristic importance is a unitless number which was used to rank the variables based on their contribution in the classification by SVM model. This was calculated as the square of the weight calculated in the SVM model for each variable with the following Equation (4):

(4) $$Imporatnce=w^{2}={\left(\overset{k=N}{\underset{k=1}{\sum }}\alpha_{k}x_{k}l_{k}\right)}^{2}$$

where $w^{2}$ gives the importance score and it is the entry wise square of the weight for each gait variable in the model. $\alpha_{k}$ represents the model parameter trained on data $\left\{x_{k},l_{k}\right\}$, where *k* is 1 to *N*. *N* represents the sample size, $x_{k}$ is each subject data with corresponding label $l_{k}$. For ML, standard commands for SVM with different kernels (RBF and linear) and default parameters (slack variable-C:1) were used from SciKit-learn library in Python [44] for comparison among walking protocols and gait assessment systems. Similarly in RF, 100 trees were used for final performance estimation.

## 3. Results

Demographic characteristics are shown in Table 1. Compared to HCs, PDs had comparable height, weight, and BMI, included proportionally more males; were significantly younger; presented with significantly lower balance confidence (ABC) and poorer cognition (MMSE). Mostly PDs were at mild to moderate stage of the diseases based on the Hoehn & Yahr scale. PD gait was assessed within 23.8 months of clinical diagnosis while taking average 398 mg/day LEDD.

Table 2 shows the main effects and interaction effect for the group (PD vs. HC), walking protocols and gait assessment systems on gait characteristics. Table 3 shows the mean and standard deviation of raw gait characteristics and the statistical difference for each normalized gait characteristic between PD and HC for the two walking tasks (IW and CW) and two gait assessment systems (GAITRite vs. Axivity). The results for the multi regression normalization are given in the supplementary Tables S1 and S2. Plots of the whole data set to check the distribution, outliers, confidence intervals, and AUC are shown in Figure S1 in the Supplementary Material. Correlations among the gait characteristics are given in Supplementary Figure S2. Gait characteristics were categorized into five domains (pace, rhythm, variability, asymmetry, and postural control) [11] based on a model of gait to help summarize findings.

Firstly, we established the effect of protocol and sensor system on gait characteristics as a first step to evaluate ML performance. There were significant main and interaction effects of pathology, walking protocols, and gait assessment systems on gait as shown in Table 2 and the individual gait characteristics are displayed in Table 3.

For group (PD vs. HC) people with PD had worse gait performance compared to controls irrespective of the protocol or gait assessment system. Grouping variables by domain [11], in general PD pace and rhythm were significantly slower while variability and asymmetry were higher, in both IW and CW protocols for both gait assessment systems.

There was a main effect of walking protocol (IW & CW) on gait characteristics. Performance was typically greater (higher pace, rhythm, variability, and asymmetry) in the IW protocol compared to the CW protocol for both PD and HC. Similarly, there were significant main and interaction effects of assessment systems on gait characteristics. In general, the values from Axivity tended to be higher compared to GAITRite although only asymmetry was significantly different between the systems.

Table 4 shows the contribution of gait characteristics in the classification modelling. A higher importance score indicates a greater contribution of each gait characteristic in the overall classification model. The top 5 Axivity characteristics were from variability, rhythm, and pace domains for both CW and IW. For GAITRite, CW contained gait characteristics from pace, rhythm and asymmetry domains, while for IW pace, rhythm and variability were important. Results without gait normalization are presented in the supplementary Table S3 and Figure S3.

Both models (SVM-RBF & RF) behaved in the similar manner for both walking protocols and gait assessment systems, with better performance of Axivity compared to GAITRite in both walking tasks with RF. Overall, SVM-RBF performed better than RF. Therefore for comparison of walking protocols and gait assessment, we only reported the results from SVM-RBF. The results of RF are given in the supplementary Table S4.

**Figure 2:**
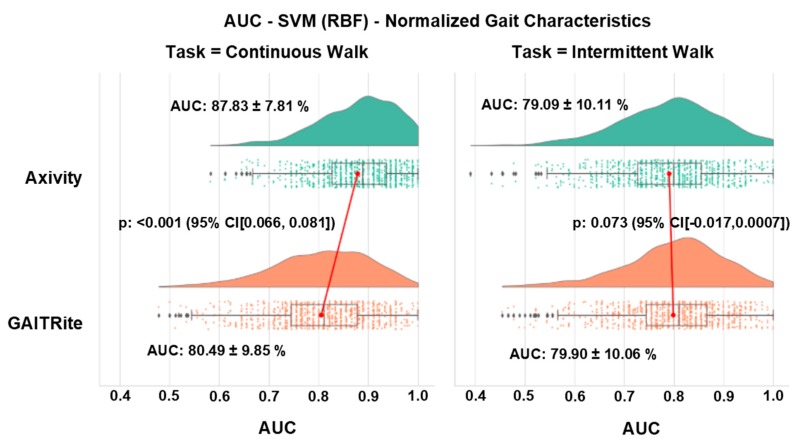
Distribution of SVM classification performance after normalization of gait characteristics.

Overall, the classification of PD was significantly more accurate with Axivity (<0.001) during the CW test (AUC 87.83 ± 7.81% for Axivity and 80.49 ± 9.85% for GAITRite), while there was no difference (*p* = 0.073) between the systems during the IW test (AUC resulted being 79.09 ± 10.11% for Axivity and 79.90 ± 10.06% for GAITRite) (Figure 2 shows the distribution of the model classification performance, where the x-axis represents the classification performance (AUC), the top x-axis represents walking protocols, and the y-axis represents the gait assessment system). For reference, SVM performance results without gait normalization are presented in the Supplementary Figure S4.

## 4. Discussion

To the best of our knowledge this is the first study to investigate the impact of different walking protocols and gait assessment systems on the performance of ML models for classification of PD. Robust normalization techniques were carried out to reduce the effect of demographics and speed on between participant differences within each group (PD and HC). A comprehensive group of 14 gait characteristics were selected based on a validated gait model. Finally, widely used SVM-RBF and RF models were trained for classification of PD and HC. The results show that different walking protocols and gait assessment systems significantly affect gait characteristics and in turn the performance of ML models. Harmonizing methods across multiple levels for comparative purposes is strongly advised to optimize and implement ML in disease classification.

### 4.1. ML Performance: An Overview

In this study, we found that the combination of CW protocol and Axivity gave the highest PD classification performance. In terms of protocols, ML performance was higher during CW with respect to IW. In terms of systems, Axivity showed a significantly higher AUC (87.83 ± 7.81%) compared to GAITRite (80.49 ± 9.85%) during CW. Similar pattern in results was achieved with RF, where Axivity showed better results compared to GAITRite during both CW & IW. Therefore, walking protocols and gait assessment systems materially impact on ML performance, which makes the comparison of previous ML studies inconclusive. In fact, previous literature has shown that, when using wearables to quantify gait, studies using 2 min CW protocols [18,19] achieved better results compared to those using 10m IW protocols [13,45]. In addition, studies showed that ML models derived from wearable inertial and force feet sensors [14,19,45,46] performed relatively better when compared to studies based on GAITRite data [17].

It’s important to underline that many factors can influence ML results: not only walking protocols and gait assessment systems, but also cohort size, disease severity stage of PD, and validation method. However, in the context of this study, we showed that walking protocol and gait assessment have a significant impact on ML performance.

### 4.2. Effect of Walking Protocols on ML Model and Performance

In general, ML performance was higher during CW with respect to IW. There are a number of possible explanations for this. The gait characteristics included in each ML model were different for CW and IW, which may explain the differences in classification performances (i.e., CW higher AUC than IW). Indeed performance of ML models are influenced by the characteristics included in the model and those characteristics are in turn influenced by the protocol used to assess gait. During IW we observed higher gait performance (e.g., higher pace, etc.) for all characteristics and for both groups (HC and PD). Acceleration and deceleration phases at the beginning and end of each IW increase the dispersion of gait characteristics, especially variability and asymmetry compared to CW where gait was sampled under more steady state conditions (lower variability and asymmetry values for both walking systems).

Another aspect is that even though participants were instructed to walk at their normal preferred pace for both protocols, it is clear that gait performance was faster during the IW compared to CW, and this has been reported previously [21]. The reasons for this are most likely because attention to performance is higher during short intermittent walking tests than a continuous steady state—where walking is performed with less attention and conscious effort. However this will also influence the dispersion of gait characteristics for PD and HC as seen by the standard deviations from Table 3. For accurate classification between groups, this dispersion within each group for each characteristic should be minimized to increase the distance between groups. This explains the need to overcome between subject variability within each group to enhance ML performance.

Collectively, this suggests that the walking protocol should be selected carefully and protocols that capture more steady state gait (in our case CW) may be optimal for classification and therefore early identification of PD.

### 4.3. Effect of Gait Assessment Systems on ML Model and Performance

In general, Axivity showed significantly higher classification performance compared to GAITRite during CW and comparable performance during IW. Gait characteristics quantified by the two systems showed significant differences. Even if these two systems (GAITRite and Axivity) measured the same spatial-temporal characteristics from the same walking tasks, the mechanism by which gait characteristics are derived is different. GAITRite determines footfalls based on pressure sensors that identify each step from which additional gait characteristics are derived [47]. Axivity, instead, uses accelerometers which detect movement continuously: individual characteristics are then derived from the raw signal. In previous work comparing gait characteristics from Axivity and GAITRite, mean spatiotemporal gait characteristics (such as walking speed, step length and step time) showed high agreement, while variability and asymmetry showed low agreement between the systems [10]. Gait characteristics extracted from GAITRite are more variable (wider dispersion) at slow speed [48]. Conversely, an accelerometer positioned at lower back may mis-detect gait events like initial and final contacts which may impact on gait characteristic quantification [49]. An accelerometer close to the center of mass of the body can capture small variations in body movement (variability) during walking [50] with higher sensitivity compared to GAITRite. Analysis of the current study indicated that Axivity is more sensitive to detect variability and asymmetry, particularly in PD. Collectively all these factors most likely influence: (i) the observed differences in the gait characteristics quantified by each system and (ii) ultimately the performance of the ML models.

**Figure 3:**
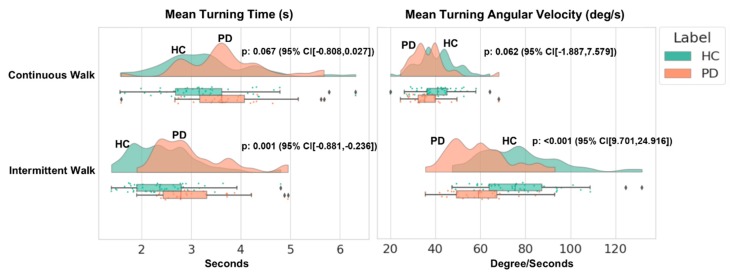
Distribution of turning characteristics.

The highest classification performance obtained with Axivity during the CW could also simply be due to the higher amount of data available for Axivity vs. GAITRite during the walking protocol: Axivity continuously sampled the entire 2 min while GAITRite sampled only each pass over the mat (e.g., four passes) during the same time frame. This is in part corroborated by the fact that we found comparable performances during the IW, when the amount of data was similar for both the systems. A further explanation for the difference in performance could be related to the inclusion of gradual turns with Axivity during the CW protocol which could have influenced the ML model. Axivity is not able to quantify turning due to the lack of a gyroscope; to try and address this, we measured turning from a sensor with an embedded gyroscope (Opal Mobility lab system APDM Inc., Portland, OR, USA) collected concurrently in a subsample of the same cohort (PD: 31, HC: 49) during CW and IW (Figure 3 shows the probability distribution of turning gait characteristics, where the x-axis represents the corresponding units and the y-axis represents the walking protocols). Turning time and angular velocity were significantly different during IW and so the turning segment of the signal was removed from Axivity analysis to delineate the gait characteristics. There were no significant between-group differences in turning characteristics during CW, and so step data from the turning component was retained for the analysis. However, we can’t rule out the possibility that steps from the turning component during CW may have contributed to better classification between groups when using Axivity.

### 4.4. Effect of Gait Normalization on ML Performance

From our results, it is clear that participants walked faster during the IW as compared to CW with both gait assessment systems (Table 3). This higher step velocity acts as a function of other gait characteristics [28,29,30], which can be influenced by its high variability. To find the appropriate walking protocol, multiple regression (MR) normalization using demographics and gait speed was important to reveal important influencing variables and overcome between participant differences among groups. Our findings support this approach, in fact we found that by controlling the effect of speed and demographics, SVM was able to differentiate between PD and HC more accurately. SVM performance increased by 5–7% in CW and 5–9% in the IW in both gait assessment systems (Supplementary Figure S4). The results are in line with previous work where similar gait normalization approaches have been used for better classification [16,31]. Thus, in short walks (IW), normalization may act as a standardized technique to overcome the effect of gait assessment systems. During CW, the effect of gait assessment systems was still significant. Normalization was also important to improve performance of ML—irrespective of protocol or system. This means that, normalization may be important to ensure standardization of walking protocol and gait assessment systems for optimal ML performance.

### 4.5. Limitations

This study had some limitations. Only two widely used ML models (SVM & RF) [13,14,15,16,17,18,19] were used in this study to compare the effect of walking protocols and gait assessment systems. However, future work should explore other classification models such as logistic regression and neural networks. Turning features were not included in this work due to the use of an accelerometer. In order to harmonize gait characteristics, step width and step width variability and a range of time series and frequency based characteristics were not included in the analysis because they could not be calculated from both systems. The inclusion of these additional variables may improve classification for respective systems and should be explored in future studies to investigate their impact on ML models. PD were assessed within 23.8 ± 4.2 months from clinical diagnosis, which is considered relatively early disease. The cohort assessed in this study was relatively young and results may not be applicable or generalizable to older, frailer people with PD with multi-morbidity.

## 5. Clinical Implications

Based on this study, walking protocols (IW & CW) and gait assessment systems had significant impact on the ML model performance. The extracted characteristics in CW with Axivity gave the highest performance in the classification ML model. Our work emphasizes the importance of the use of standardized walking protocols and wearable devices for ML PD classification purposes, to support clinical decision making. With the recent advancements in this field, this study will help clinicians to understand and select the appropriate walking protocols and gait assessment systems for optimal PD diagnosis. In future studies, such as those looking at prodromal disease, CW assessed with Axivity may give a more accurate reflection of gait changes. For better results, it is recommended to control for demographics and walking speed for gait characteristics normalization in the PD ML classification modeling. Intervention studies seeking to determine changes in particular gait characteristic(s) may be advised to use this methodology.

## 6. Conclusions

In this study, the impact of different walking protocols (CW & IW) and gait assessment systems (GAITRite & Axivity) on the performance of widely used ML models SVM and RF was investigated. Gait characteristics were normalized with respect to demographic properties and walking speed to overcome the between participants’ differences within each group (HC and PD) for each walking protocol (CW vs. IW). Both ML models behaved in similar fashion for both walking protocols and gait assessment systems. Higher performances were achieved with CW compared to IW. Axivity gave higher classification performance compared to GAITRite. The highest PD classification performance was obtained during CW with Axivity (87.83 ± 7.81%). This work supports the idea that direct comparison of various ML studies using different walking protocols and gait assessment systems may not be appropriate. The findings from this study suggest that the choice of the testing protocol and gait assessment systems is important to achieve best classification results, which may have a direct impact on future end points in intervention studies. In conclusion, there is a need for standardization of walking protocols and gait assessment systems for wide scale implementation in clinical gait assessment.

## Figures and Tables

**Table 1:** Demographic and clinical characteristics of the participants.

Demographics	HC (n = 103) Mean ± SD	PD (n = 93) Mean ± SD	*p*
M/F	49/54	59/34	**0.026**
Age (years)	72.3 ± 6.7	69.2 ± 10.1	**0.012**
Height (m)	1.7 ± 0.09	1.7 ± 0.09	0.623
Mass (kg)	78.6 ± 14.3	78.6 ± 15.9	0.999
BMI (kg/m²) ^1^	27.2 ± 5.6	27.5 ± 4.7	0.750
MMSE (0–30) ^2^	28.9 ± 1.9	28.4 ± 1.6	0.102
ABCs (0–100)% ^3^	91.2 ± 13.8	80.6 ± 20.7	**<0.001**
LEDD, mg/day ^4^		397.7 ± 217.2	
Disease Duration (months)		23.8 ± 4.2	
Hoehn & Yahr (n)		HY I: 8	
		HY II: 71	
		HY III: 14	
MDS-UPDRS III ^5^		32.4 ± 10.3	
		(HY I: 17.4 ± 4.5)	
		(HY II: 32.9 ± 9.7)	
		(HY III: 38.1 ± 7.5)	
Motor Phenotype (n)		^6^ PIGD: 34	
		^7^ ID: 16	
		^8^ TD: 43	

^1^ BMI: Body Mass Index; ^2^ MMSE: Mini–Mental State Examination; ^3^ ABC: Activities specific balance confidence scale; ^4^ LEDD: Levodopa equivalent daily dose; ^5^ MDS-UPDRS III: Movement Disorders Unified Parkinson’s Disease Rating Scale part III; ^6^ PIGD: Postural instability and gait disorder phenotype; ^7^ ID: Indeterminate phenotype; ^8^ TD: Tremor dominant phenotype. In bold significant *p*-values (*p* < 0.05).

**Table 2:** MANOVA to check the effect of walking protocols and gait assessment systems on gait (* indicates interaction).

Effect Assessment on Gait	MANOVA		
	Wilk’s Lambda	F	*p*-Value
Group (HC & PD)	0.803	14.198	<0.001
Walking Protocols	0.463	67.337	<0.001
Gait Assessment Systems	0.067	805.792	<0.001
Group * Protocol	0.949	3.092	<0.001
Group * Systems	0.853	9.991	<0.001
Protocols * Systems	0.513	55.168	<0.001

**Table 3:** Mean comparison among PD and HC for gait characteristics obtained from walking protocols and assessment systems (significant normalized gait characteristics are highlighted in grey color, in bold significant *p*-values (*p* < 0.05) for normalized gait characteristics except step velocity).

Gait Domains	Gait Characteristics	Intermittent Walk (IW)			Continuous Walk (CW)		
		HC (n = 103) Mean ± SD	PD (n = 93) Mean ± SD	*p* Value	HC (n = 103) Mean ± SD	PD (n = 93) Mean ± SD	*p* Value
**Gait Characteristics from Axivity**							
Pace	Step Velocity (m/s)	1.324 ± 0.153	1.252 ± 0.226	**0.002**	1.283 ± 0.155	1.186 ± 0.262	**0.009**
	Step Length (m)	0.718 ± 0.094	0.717 ± 0.074	**0.010**	0.694 ± 0.121	0.690 ± 0.077	**0.022**
	Swing Time Variability (s)	0.064 ± 0.084	0.123 ± 0.144	**<0.001**	0.037 ± 0.031	0.108 ± 0.082	0.058
Rhythm	Step Time (s)	0.554 ± 0.052	0.614 ± 0.129	**0.001**	0.538 ± 0.046	0.609 ± 0.133	**<0.001**
	Swing Time (s)	0.394 ± 0.047	0.448 ± 0.116	**0.001**	0.386 ± 0.044	0.454 ± 0.125	**<0.001**
	Stance Time (s)	0.705 ± 0.059	0.767 ± 0.141	**0.003**	0.689 ± 0.054	0.763 ± 0.144	**<0.001**
Variability	Step Velocity Variability (m/s)	0.174 ± 0.097	0.196 ± 0.078	0.273	0.137 ± 0.060	0.190 ± 0.076	**<0.001**
	Step Length Variability (m)	0.101 ± 0.060	0.126 ± 0.059	**0.022**	0.072 ± 0.034	0.109 ± 0.044	**<0.001**
	Step Time Variability (s)	0.093 ± 0.103	0.162 ± 0.157	**<0.001**	0.037 ± 0.033	0.114 ± 0.087	**<0.001**
	Stance Time Variability (s)	0.094 ± 0.103	0.166 ± 0.158	**0.001**	0.039 ± 0.033	0.116 ± 0.088	**<0.001**
Asymmetry	Step Time Asymmetry (s)	0.031 ± 0.018	0.051 ± 0.034	0.610	0.021 ± 0.016	0.026 ± 0.025	0.268
	Swing Time Asymmetry (s)	0.023 ± 0.017	0.039 ± 0.028	0.437	0.020 ± 0.018	0.023 ± 0.024	0.592
	Stance Time Asymmetry (s)	0.030 ± 0.019	0.044 ± 0.027	0.771	0.020 ± 0.018	0.024 ± 0.02	0.419
Postural Control	Step length Asymmetry (m)	0.078 ± 0.053	0.119 ± 0.112	0.606	0.066 ± 0.052	0.126 ± 0.128	0.060
**Gait Characteristics from GAITRite**							
Pace	Step Velocity (m/s)	1.338 ± 0.198	1.194 ± 0.223	**<0.001**	1.301 ± 0.192	1.135 ± 0.218	**<0.001**
	Step Length (m)	0.697 ± 0.084	0.636 ± 0.098	**<0.001**	0.683 ± 0.083	0.616 ± 0.097	**<0.001**
	Swing Time Variability (s)	0.013 ± 0.003	0.016 ± 0.008	**0.327**	0.013 ± 0.004	0.017 ± 0.009	**0.010**
Rhythm	Step Time (s)	0.525 ± 0.045	0.538 ± 0.047	**<0.001**	0.528 ± 0.044	0.548 ± 0.047	**<0.001**
	Swing Time (s)	0.385 ± 0.030	0.382 ± 0.033	**<0.001**	0.385 ± 0.029	0.384 ± 0.031	**0.001**
	Stance Time (s)	0.665 ± 0.068	0.695 ± 0.072	**<0.001**	0.674 ± 0.066	0.714 ± 0.074	**<0.001**
Variability	Step Velocity Variability (m/s)	0.051 ± 0.015	0.047 ± 0.014	0.946	0.050 ± 0.012	0.054 ± 0.014	**0.005**
	Step Length Variability (m)	0.019 ± 0.006	0.020 ± 0.007	**0.008**	0.020 ± 0.006	0.023 ± 0.007	0.338
	Step Time Variability (s)	0.014 ± 0.004	0.016 ± 0.007	0.173	0.014 ± 0.004	0.018 ± 0.006	**0.018**
	Stance Time Variability (s)	0.016 ± 0.005	0.019 ± 0.011	0.260	0.017 ± 0.006	0.023 ± 0.012	**0.011**
Asymmetry	Step Time Asymmetry (s)	0.011 ± 0.008	0.018 ± 0.018	**0.003**	0.012 ± 0.009	0.019 ± 0.022	**0.007**
	Swing Time Asymmetry (s)	0.007 ± 0.006	0.014 ± 0.014	**<0.001**	0.007 ± 0.006	0.014 ± 0.014	**0.003**
	Stance Time Asymmetry (s)	0.007 ± 0.006	0.014 ± 0.014	0.476	0.007 ± 0.006	0.015 ± 0.015	**<0.001**
Postural Control	Step length Asymmetry (m)	0.020 ± 0.016	0.022 ± 0.018	**0.048**	0.019 ± 0.015	0.022 ± 0.020	**0.036**

**Table 4:** Importance of normalized gait characteristics in the classification of PD.

Sensing System	Intermittent Walk		Continuous Walk	
	Characteristic	Importance	Characteristic	Importance
Axivity	Mean Step Length	0.22	Step Velocity Variability	1.10
	Mean Stance Time	0.20	Mean Swing Time	0.72
	Mean Swing Time	0.15	Mean Step Length	0.49
	Swing Time Variability	0.14	Stance Time Variability	0.20
	Mean Step Time	0.07	Step Length Variability	0.12
GAITRite	Mean Step Time	0.23	Mean Step Length	3.80
	Step Velocity Variability	0.22	Mean Step Time	2.72
	Step Length Variability	0.15	Stance Time Asymmetry	1.21
	Swing Time Variability	0.14	Mean Stance Time	1.10
	Mean Step Length	0.09	Swing Time Asymmetry	0.72

## Supplementary Materials

The following are available online at https://www.mdpi.com/1424-8220/19/24/5363/s1, Table S1: Correlation of gender, age, BMI, and step velocity (speed) with gait characteristics before and after normalization, Table S2: Coefficients from the regression model by using the healthy control participants, Table S3: Importance of gait characteristics in the classification of PD before and after gait characteristics normalization, Table S4: Random forest (RF) classification performance after gait normalization, Figure S1: Distribution of the gait characteristics from 5 domains of conceptual gait model with statistical analysis, Figure S2: Correlations among gait characteristics before and after normalization, Figure S3: Contribution of the gait characteristics in the classification modelling in Support Vector Machine, Figure S4: SVM performance before gait characteristics normalization.
