# Supplementary material for: Comparison of Walking Protocols and Gait Assessment Systems for Machine Learning-Based Classification of Parkinson’s Disease
Source: Sensors (Basel). 2019 Dec 5;19(24):5363. doi: 10.3390/s19245363 (PMC6960714; doi:10.3390/s19245363)

Comparison of Walking Protocols and Gait Assessment Systems for Machine Learning-Based Classification of Parkinson's Disease

Rana Zia Ur Rehman [1], Silvia Del Din [1], Jian Qing Shi [2], Brook Galna [1,3], Sue Lord [1,4], Alison J. Yarnall [1,5], Yu Guan [6] and Lynn Rochester [1,5,*]

[1] Institute of Neuroscience/Institute for Ageing, Newcastle University, Newcastle Upon Tyne NE4 5PL, UK; rana.zia-ur-rehman@ncl.ac.uk (R.Z.U.R.); silvia.del-din@ncl.ac.uk (S.D.D.); brook.galna@newcastle.ac.uk (B.G.); sue.lord@aut.ac.nz (S.L.); alison.yarnall@ncl.ac.uk (A.J.Y.)
[2] School of Mathematics, Statistics, and Physics, Newcastle University, Newcastle Upon Tyne NE1 7RU, UK; jian.shi@ncl.ac.uk
[3] School of Biomedical, Nutritional and Sport Sciences, Newcastle University, Newcastle Upon Tyne NE1 7RU, UK
[4] Department of Physiotherapy, Auckland University of Technology, 92006 Auckland, New Zealand
[5] The Newcastle upon Tyne Hospitals NHS Foundation Trust, Newcastle Upon Tyne NE7 7DN, UK
[6] School of Computing, Newcastle University, Newcastle Upon Tyne, NE4 5TG, UK; yu.guan@ncl.ac.uk
* Correspondence: lynn.rochester@ncl.ac.uk; Tel.: +44-(0)-191-208-1291; Fax: +44-(0)-191-208-1251

Supplementary Material

Table S1. Correlation of gender, age, BMI, and step velocity (speed) with gait characteristics before and after normalization.

Spearson's correlations	System	Walk Type	Data Type	Gender	Age	BMI	Step Velocity(Speed)
Mean Step Time	Axivity	Continuous	Raw	-.338 (<.001)	-.045 (.534)	.108 (.131)	-.506 (<.001)
			Normalized	.117 (.102)	.083 (.250)	-.070 (.333)	.106 (.141)
	GAITRite	Continuous	Raw	-.445 (<.001)	-.030 (.672)	.073 (.309)	-.545 (<.001)
			Normalized	.183 (.010)	.162 (.023)	-.058 (.418)	.071 (.323)
	Axivity	Intermittent	Raw	-.324 (<.001)	-.087 (.227)	.155 (.030)	-.404 (<.001)
			Normalized	.109 (.128)	-.039 (.589)	-.019 (.792)	-.029 (.687)
	GAITRite	Intermittent	Raw	-.457 (<.001)	-.035 (.624)	.079 (.270)	-.564 (<.001)
			Normalized	.208 (.003)	.147 (.040)	-.043 (.551)	.055 (.447)
Mean Stance Time	Axivity	Continuous	Raw	-.392 (<.001)	-.034 (.639)	.080 (.266)	-.457 (<.001)
			Normalized	.135 (.060)	.048 (.508)	-.041 (.571)	.075 (.297)
	GAITRite	Continuous	Raw	-.415 (<.001)	.029 (.689)	.197 (.006)	-.686 (<.001)
			Normalized	.142 (.047)	.059 (.413)	-.035 (.627)	.034 (.638)
	Axivity	Intermittent	Raw	-.360 (<.001)	-.074 (.304)	.136 (.057)	-.369 (<.001)
			Normalized	.099 (.169)	-.063 (.379)	.029 (.690)	-.039 (.585)
	GAITRite	Intermittent	Raw	-.425 (<.001)	.022 (.755)	.187 (.009)	-.706 (<.001)
			Normalized	.137 (.055)	.059 (.409)	-.017 (.810)	.022 (.758)
Mean Swing Time	Axivity	Continuous	Raw	-.232 (.001)	-.047 (.516)	.163 (.022)	-.506 (<.001)
			Normalized	.061 (.396)	-.063 (.380)	.041 (.573)	.061 (.397)
	GAITRite	Continuous	Raw	-.389 (<.001)	-.200 (.005)	-.218 (.002)	-.006 (.929)
			Normalized	.100 (.164)	-.073 (.308)	-.211 (.003)	.087 (.226)
	Axivity	Intermittent	Raw	-.277 (<.001)	-.039 (.587)	.205 (.004)	-.456 (<.001)
			Normalized	.074 (.305)	.023 (.745)	.029 (.692)	-.025 (.732)
	GAITRite	Intermittent	Raw	-.393 (<.001)	-.183 (.010)	-.190 (.008)	-.062 (.387)
			Normalized	.129 (.071)	-.059 (.415)	-.188 (.008)	.049 (.499)
Mean Step Length	Axivity	Continuous	Raw	-.167 (.019)	-.226 (.001)	-.089 (.213)	.762 (<.001)
			Normalized	.144 (.045)	.085 (.234)	-.065 (.368)	.120 (.093)
	GAITRite	Continuous	Raw	-.213 (.003)	-.275 (<.001)	-.216 (.002)	.852 (<.001)
			Normalized	.162 (.024)	.008 (.911)	-.087 (.227)	.282 (<.001)
	Axivity	Intermittent	Raw	-.127 (.076)	-.229 (.001)	-.013 (.853)	.767 (<.001)
			Normalized	.159 (.026)	-.083 (.247)	.048 (.507)	.077 (.286)
	GAITRite	Intermittent	Raw	-.217 (.002)	-.290 (<.001)	-.189 (.008)	.811 (<.001)
			Normalized	.166 (.020)	-.028 (.701)	-.057 (.424)	.225 (.002)
Step Time Variability	Axivity	Continuous	Raw	-.065 (.366)	.104 (.146)	.002 (.981)	-.174 (.014)
			Normalized	.028 (.701)	.051 (.479)	-.080 (.265)	.132 (.064)
	GAITRite	Continuous	Raw	-.079 (.272)	.160 (.025)	.136 (.057)	-.730 (<.001)
			Normalized	.013 (.854)	-.183 (.010)	-.176 (.014)	.028 (.696)
	Axivity	Intermittent	Raw	.022 (.762)	.064 (.369)	.080 (.268)	-.009 (.899)
			Normalized	-.082 (.251)	-.205 (.004)	.044 (.543)	-.098 (.172)
	GAITRite	Intermittent	Raw	-.068 (.345)	.279 (<.001)	.131 (.068)	-.649 (<.001)
			Normalized	-.009 (.906)	-.023 (.746)	-.017 (.818)	-.082 (.250)
Stance Time Variability	Axivity	Continuous	Raw	-.062 (.391)	.088 (.222)	.006 (.929)	-.193 (.007)
			Normalized	-.012 (.864)	.027 (.711)	-.089 (.214)	.156 (.029)
	GAITRite	Continuous	Raw	-.103 (.151)	.087 (.226)	.143 (.045)	-.711 (<.001)
			Normalized	-.131 (.068)	-.164 (.022)	-.117 (.101)	-.033 (.646)
	Axivity	Intermittent	Raw	.024 (.737)	.065 (.368)	.080 (.267)	-.018 (.801)
			Normalized	-.082 (.254)	-.198 (.005)	.069 (.333)	-.075 (.298)
	GAITRite	Intermittent	Raw	-.044 (.538)	.202 (.004)	.109 (.127)	-.700 (<.001)
			Normalized	.089 (.216)	.031 (.663)	-.019 (.794)	-.234 (.001)
Swing Time Variability	Axivity	Continuous	Raw	-.050 (.488)	.093 (.195)	.013 (.860)	-.236 (.001)
			Normalized	.026 (.713)	.184 (.010)	.002 (.981)	-.004 (.950)
	GAITRite	Continuous	Raw	-.142 (.047)	.220 (.002)	.097 (.178)	-.667 (<.001)
			Normalized	-.099 (.167)	-.062 (.386)	-.055 (.443)	-.098 (.172)
	Axivity	Intermittent	Raw	.022 (.762)	.067 (.352)	.116 (.107)	-.187 (.008)
			Normalized	-.126 (.078)	-.117 (.101)	.004 (.958)	-.153 (.033)
	GAITRite	Intermittent	Raw	-.201 (.005)	.377 (<.001)	.155 (.031)	-.609 (<.001)
			Normalized	.141 (.049)	.155 (.030)	.009 (.901)	.407 (<.001)
Step Length Variability	Axivity	Continuous	Raw	.096 (.180)	.026 (.713)	.035 (.624)	.127 (.076)
			Normalized	.021 (.766)	-.116 (.106)	.091 (.203)	-.116 (.107)
	GAITRite	Continuous	Raw	-.199 (.005)	.343 (<.001)	-.017 (.812)	-.376 (<.001)
			Normalized	-.213 (.003)	.034 (.638)	.108 (.132)	-.174 (.015)
	Axivity	Intermittent	Raw	.119 (.097)	.007 (.918)	.099 (.167)	.246 (.001)
			Normalized	-.002 (.980)	-.062 (.385)	.082 (.254)	-.259 (<.001)
	GAITRite	Intermittent	Raw	-.081 (.258)	.326 (<.001)	.150 (.036)	-.390 (<.001)

			Normalized	.220 (.002)	-.346 (<.001)	-.210 (.003)	.673 (<.001)
Step Velocity Variability	Axivity	Continuous	Raw	.183 (.010)	.012 (.873)	-.003 (.967)	.312 (<.001)
			Normalized	.002 (.980)	-.152 (.034)	.118 (.099)	-.158 (.027)
	GAITRite	Continuous	Raw	-.019 (.793)	.112 (.118)	-.097 (.177)	-.075 (.299)
			Normalized	-.401 (<.001)	.133 (.064)	-.040 (.575)	-.107 (.137)
	Axivity	Intermittent	Raw	.191 (.007)	.014 (.846)	.050 (.490)	.359 (<.001)
			Normalized	-.005 (.942)	-.103 (.150)	.072 (.318)	-.179 (.012)
	GAITRite	Intermittent	Raw	.128 (.073)	.139 (.053)	.004 (.953)	-.087 (.227)
			Normalized	-.197 (.006)	.195 (.006)	.074 (.300)	-.273 (<.001)
Step Time Asymmetry	Axivity	Continuous	Raw	-.084 (.243)	-.046 (.519)	.017 (.809)	-.002 (.973)
			Normalized	.047 (.515)	-.049 (.496)	-.004 (.953)	.043 (.547)
	GAITRite	Continuous	Raw	-.105 (.142)	-.090 (.209)	.030 (.680)	-.236 (.001)
			Normalized	-.058 (.421)	-.113 (.115)	.019 (.795)	-.170 (.017)
	Axivity	Intermittent	Raw	-.063 (.384)	-.014 (.842)	.057 (.426)	.044 (.540)
			Normalized	-.581 (<.001)	-.007 (.925)	.037 (.604)	.042 (.561)
	GAITRite	Intermittent	Raw	-.195 (.006)	-.087 (.225)	.142 (.046)	-.260 (<.001)
			Normalized	-.088 (.220)	-.119 (.097)	.099 (.167)	-.162 (.023)
Stance Time Asymmetry	Axivity	Continuous	Raw	-.071 (.325)	-.038 (.595)	.057 (.428)	-.043 (.550)
			Normalized	.011 (.880)	.122 (.088)	.185 (.010)	-.205 (.004)
	GAITRite	Continuous	Raw	-.135 (.060)	-.068 (.343)	.081 (.258)	-.283 (<.001)
			Normalized	-.059 (.415)	-.149 (.038)	.015 (.835)	-.163 (.023)
	Axivity	Intermittent	Raw	-.028 (.696)	-.052 (.470)	.023 (.746)	-.044 (.537)
			Normalized	.248 (<.001)	-.082 (.255)	-.113 (.114)	.324 (<.001)
	GAITRite	Intermittent	Raw	-.188 (.008)	-.023 (.745)	.173 (.015)	-.245 (.001)
			Normalized	.257 (<.001)	-.246 (.001)	.064 (.372)	.029 (.687)
Swing Time Asymmetry	Axivity	Continuous	Raw	-.055 (.440)	-.062 (.390)	.021 (.771)	-.037 (.603)
			Normalized	-.012 (.872)	.025 (.729)	.067 (.352)	.141 (.049)
	GAITRite	Continuous	Raw	-.142 (.047)	-.070 (.330)	.110 (.125)	-.351 (<.001)
			Normalized	-.043 (.550)	-.150 (.035)	.008 (.910)	-.140 (.050)
	Axivity	Intermittent	Raw	-.026 (.716)	-.015 (.839)	.019 (.797)	-.149 (.037)
			Normalized	-.006 (.934)	.134 (.061)	-.004 (.958)	-.006 (.938)
	GAITRite	Intermittent	Raw	-.212 (.003)	.002 (.975)	.127 (.076)	-.256 (<.001)
			Normalized	.348 (<.001)	-.034 (.636)	-.143 (.045)	.336 (<.001)
Step Length Asymmetry	Axivity	Continuous	Raw	-.027 (.705)	-.120 (.094)	.006 (.938)	.000 (.995)
			Normalized	.031 (.666)	-.001 (.984)	-.047 (.514)	-.085 (.234)
	GAITRite	Continuous	Raw	-.152 (.033)	.042 (.558)	.088 (.222)	-.103 (.151)
			Normalized	.242 (.001)	-.057 (.430)	-.106 (.138)	.162 (.023)
	Axivity	Intermittent	Raw	.002 (.978)	-.124 (.085)	-.109 (.128)	.350 (<.001)
			Normalized	.038 (.596)	-.158 (.027)	-.038 (.597)	.079 (.272)
	GAITRite	Intermittent	Raw	-.253 (<.001)	.094 (.190)	.126 (.077)	-.060 (.406)
			Normalized	.467 (<.001)	-.121 (.092)	-.189 (.008)	.190 (.008)

Table S2. Coefficients from the regression model by using the healthy control subjects

Regression Coefficients	Sensing System	Walk Type	Model Coefficients					Adjusted R^2	Standard Error
			β_0	β_{Gender}	β_{Age}	β_{BMI}	$\beta_{Step\ Velocity\ (Speed)}$		
Mean Step Time	Axivity	Continuous	0.873	-0.048	-0.002	0	-0.178	0.595	0.029
		Intermittent	0.774	-0.047	-0.001	0.001	-0.134	0.368	0.042
	GAITRite	Continuous	8.72E-01	-4.90E-02	-2.00E-03	-1.00E-03	-1.41E-01	0.653	0.025
		Intermittent	0.877	-0.053	-0.002	-1.00E-03	-0.138	0.691	0.025
Mean Stance Time	Axivity	Continuous	1.066	-0.064	-0.002	-0.001	-0.182	0.588	0.035
		Intermittent	0.955	-0.06	-0.001	0	-0.129	0.379	0.046
	GAITRite	Continuous	1.184	-6.70E-02	-0.002	0	-0.254	0.739	0.034
		Intermittent	1.181	-0.069	-0.002	0.00E+00	-2.46E-01	0.759	0.034
Mean Swing Time	Axivity	Continuous	0.677	-0.031	-0.001	-6.40E-05	-0.175	0.454	0.032
		Intermittent	0.597	-0.031	-0.001	0.001	-0.136	0.31	0.039
	GAITRite	Continuous	5.60E-01	-0.033	-1.00E-03	-0.001	-0.026	0.393	0.023
		Intermittent	0.568	-0.036	-0.001	-1.00E-03	-0.027	0.455	0.022
Mean Step Length	Axivity	Continuous	0.365	-0.051	-0.002	0	0.338	0.796	0.029
		Intermittent	0.293	-0.056	-0.001	0	0.372	0.783	0.034
	GAITRite	Continuous	0.474	-6.60E-02	-2.00E-03	-1.00E-03	3.29E-01	0.839	0.033
		Intermittent	0.479	-0.071	-0.002	-0.001	3.33E-01	0.84	0.034
Step Time Variability	Axivity	Continuous	0.054	-0.002	0	0	-0.027	0.139	0.033
		Intermittent	-0.156	0.012	0.002	0.001	0.041	0.028	0.103
	GAITRite	Continuous	0.023	0.00E+00	8.75E-05	6.81E-05	-0.014	0.383	0.003
		Intermittent	0.023	0	8.93E-05	-2.44E-06	-1.20E-02	0.361	0.003
Stance Time Variability	Axivity	Continuous	0.066	-1.00E-03	0	9.35E-05	-0.037	0.179	0.034
		Intermittent	-0.16	0.013	0.002	0.001	0.04	0.03	0.104
	GAITRite	Continuous	3.40E-02	1.00E-03	5.75E-05	8.95E-05	-0.019	0.309	0.005
		Intermittent	3.80E-02	-0.001	5.27E-05	0	-0.017	0.408	0.004
Swing Time Variability	Axivity	Continuous	7.40E-02	-0.001	0.00E+00	-2.87E-05	-0.056	0.045	0.031
		Intermittent	-0.113	0.017	0.001	0.002	0.017	0.033	0.084
	GAITRite	Continuous	2.30E-02	0	7.72E-05	-1.97E-05	-0.011	0.333	0.003
		Intermittent	0.019	-0.001	0	-1.54E-05	-0.011	0.448	0.002
Step Length Variability	Axivity	Continuous	-5.10E-02	6.00E-03	0.001	7.13E-05	0.056	0.035	0.033
		Intermittent	-0.18	0.006	0.001	0.001	0.121	0.088	0.057
	GAITRite	Continuous	8.00E-03	-1.00E-03	0.00E+00	6.46E-05	-7.00E-03	0.166	0.005
		Intermittent	5.88E-05	-0.001	0	0	-6.00E-03	0.181	0.005
Step Velocity Variability	Axivity	Continuous	-0.162	2.10E-02	2.00E-03	0	1.61E-01	0.16	0.055
		Intermittent	-0.327	0.025	0.002	0.002	0.257	0.174	0.088
	GAITRite	Continuous	0.018	4.00E-03	0.00E+00	0	0	0.018	0.012
		Intermittent	0.016	0.005	0	0	0.006	0.017	0.015
Step Time Asymmetry	Axivity	Continuous	3.40E-02	-0.006	-5.24E-05	-2.99E-05	-0.005	0.039	0.016
		Intermittent	-0.01	-0.007	0	0	0.013	0.016	0.018
	GAITRite	Continuous	0.043	-0.003	0	-5.43E-05	-0.01	0.026	0.009
		Intermittent	3.10E-02	-4.00E-03	0	5.29E-05	-0.008	0.057	0.007
Stance Time Asymmetry	Axivity	Continuous	2.90E-02	-0.001	0.00E+00	0.00E+00	-0.023	0.05	0.018
		Intermittent	0.027	-0.003	0	0	-0.016	0.038	0.02
	GAITRite	Continuous	0.008	-0.001	5.06E-05	6.03E-05	-0.004	0.028	0.006
		Intermittent	0.001	-2.00E-03	6.99E-05	0	-0.002	0.046	0.006
Swing Time Asymmetry	Axivity	Continuous	0.029	-1.00E-03	0.00E+00	0	-0.021	0.045	0.018
		Intermittent	0.033	0.001	0	9.68E-05	-0.028	0.051	0.016
	GAITRite	Continuous	0.014	-0.001	2.46E-05	4.92E-05	-0.007	0.051	0.006
		Intermittent	-0.002	-2.00E-03	0	0	-0.003	0.068	0.006
Step Length Asymmetry	Axivity	Continuous	-1.20E-01	0.001	0.00E+00	0.00E+00	0.159	0.175	0.047
		Intermittent	-0.186	-6.00E-03	0.001	-0.001	0.225	0.406	0.042
	GAITRite	Continuous	-0.005	-2.00E-03	0.00E+00	0	-0.003	0.035	0.015
		Intermittent	-2.00E-03	-4.00E-03	0.00E+00	5.47E-05	-0.005	0.022	0.015

Table S3. Importance of gait characteristics in the classification of PD before and after gait characteristics normalization

Sensing System – Walk	Raw Gait Characteristics		Normalized gait characteristics	
	Characteristics name	Importance	Characteristics name	Importance
Axivity – Continuous	Step Time Variability	7.84	Step Velocity Variability	1.10
	Stance Time Variability	6.76	Mean Swing Time	0.72
	Swing Time Variability	5.62	Mean Step Length	0.49
	Step Velocity Variability	3.80	Stance Time Variability	0.20
	Step Length Asymmetry	3.06	Step Length Variability	0.12
GAITRite – Continuous	Mean Step Velocity	0.81	Mean Step Length	3.80
	Mean Step Length	0.40	Mean Step Time	2.72
	Mean Step Time	0.12	Stance Time Asymmetry	1.21
	Mean Stance Time	0.03	Mean Stance Time	1.10
	Mean Swing Time	0.03	Swing Time Asymmetry	0.72
Axivity – Intermittent	Step length Asymmetry	3.61	Mean Step Length	0.22
	Stance Time Variability	3.24	Mean Stance Time	0.20
	Step Time Variability	2.89	mean Swing Time	0.15
	Swing Time Variability	1.69	Swing Time Variability	0.14
	Step Time Asymmetry	1.21	Mean Step Time	0.07
GAITRite – Intermittent	Mean Step Velocity	1.32	Mean Step Time	0.23
	Mean Step Length	0.45	Step Velocity Variability	0.22
	Step Velocity Variability	0.09	Step Length Variability	0.15
	Mean Step Time	0.03	Swing Time Variability	0.14
	Step Length Asymmetry	0.03	Mean Step Length	0.09

Table S4. Random forest (RF) classification performance after gait normalization

Sensing Systems	Walking Protocols	
	Continuous Walk	Intermittent Walk
Axivity (AX3)	83.45 ± 8.38	78.45 ± 9.97
GAITRite	69.14 ± 11.27	69.13 ± 11.62

Figure S1. Distribution of the gait characteristics from 5 domains of conceptual gait model with statistical analysis

Pace Domain

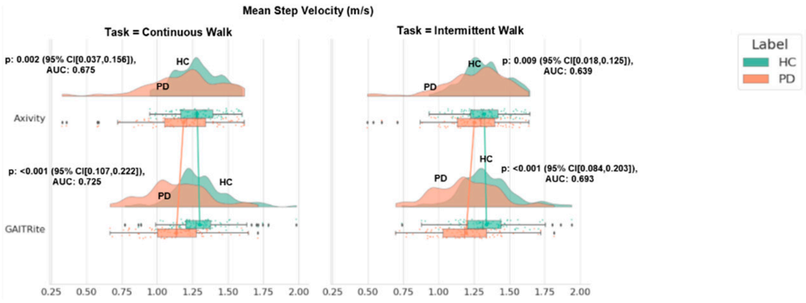

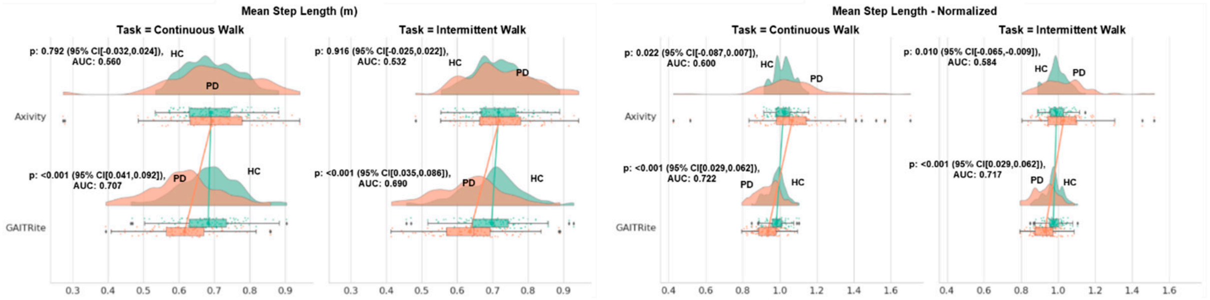

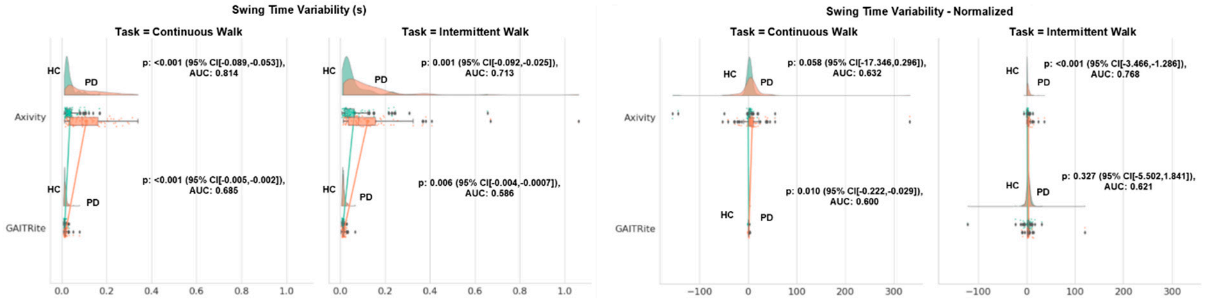

Rhythm Domain

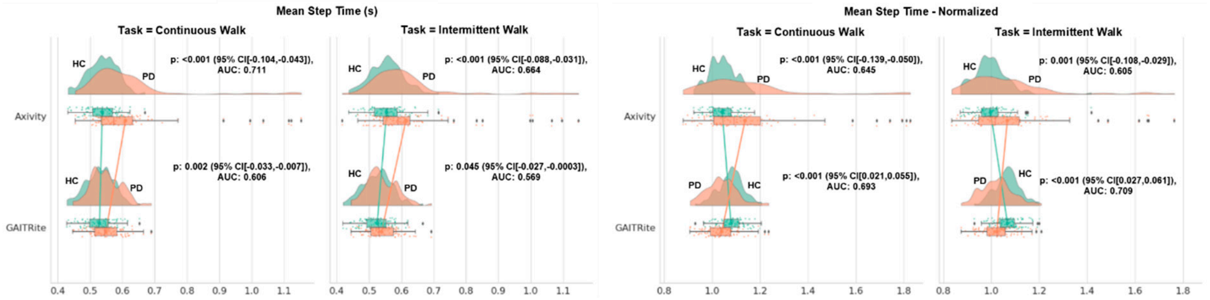

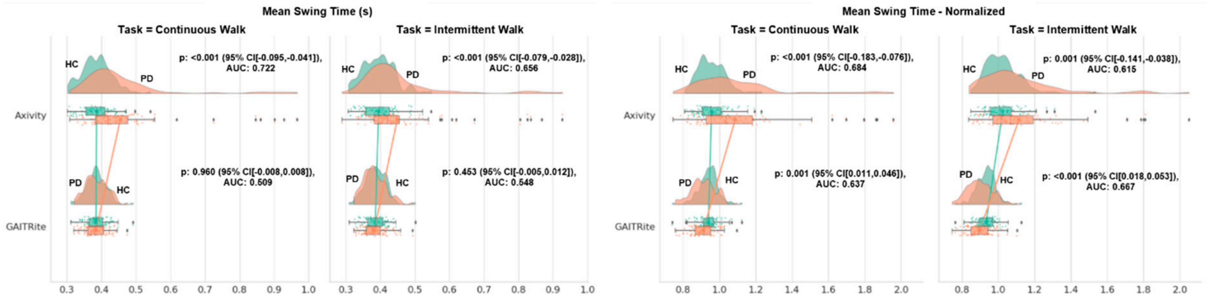

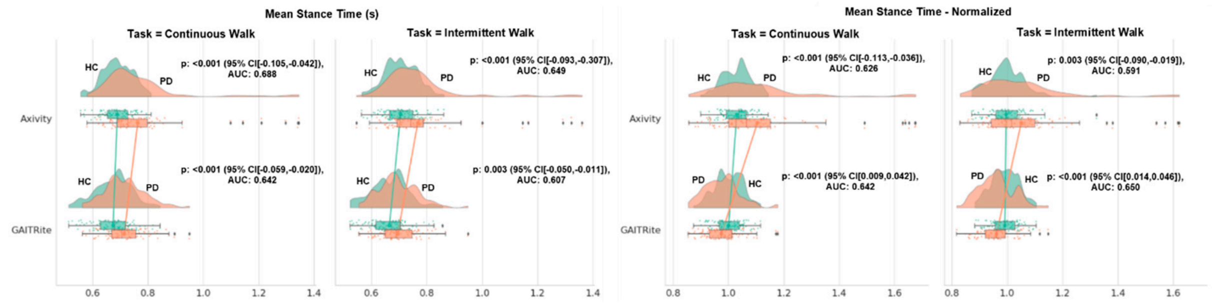

Variability Domain

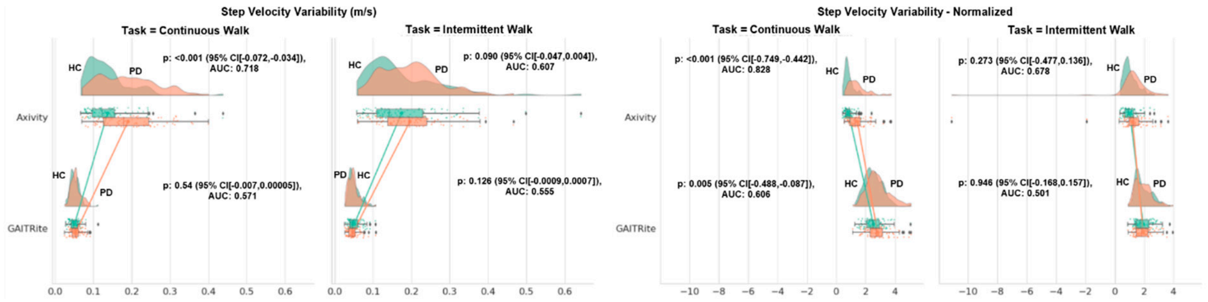

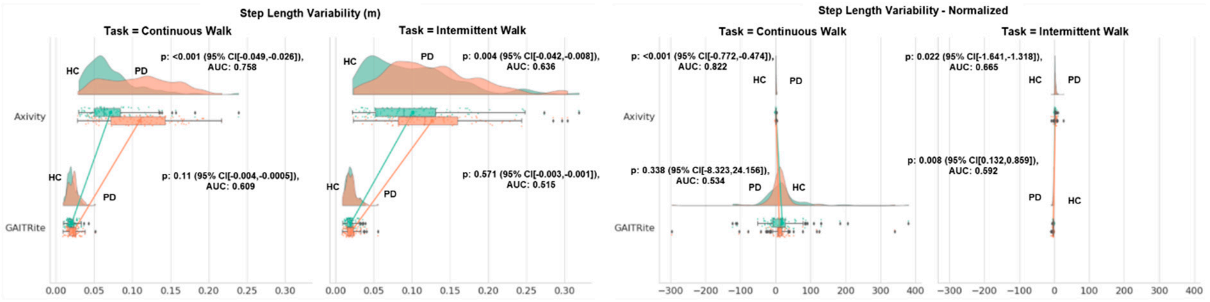

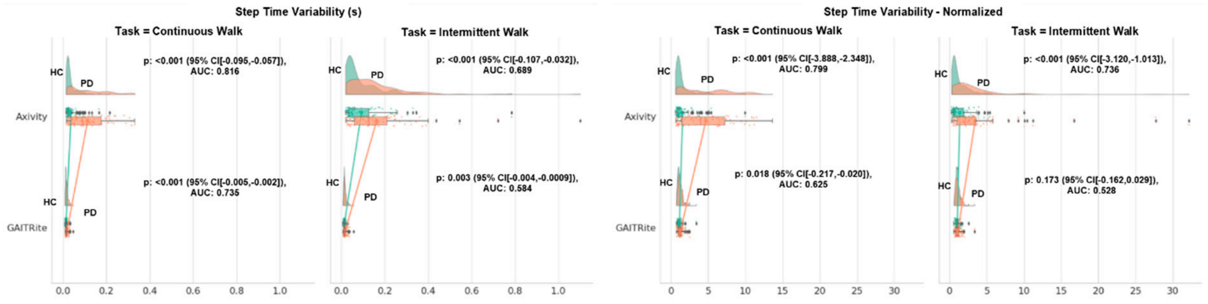

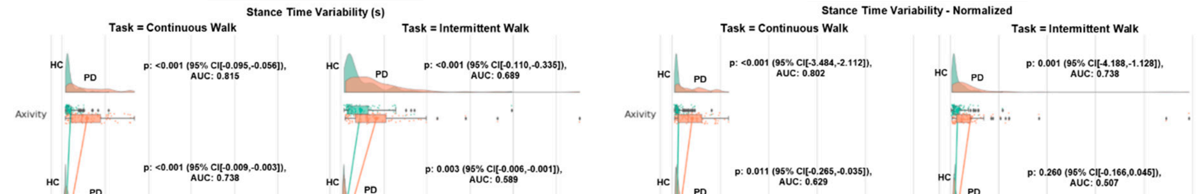

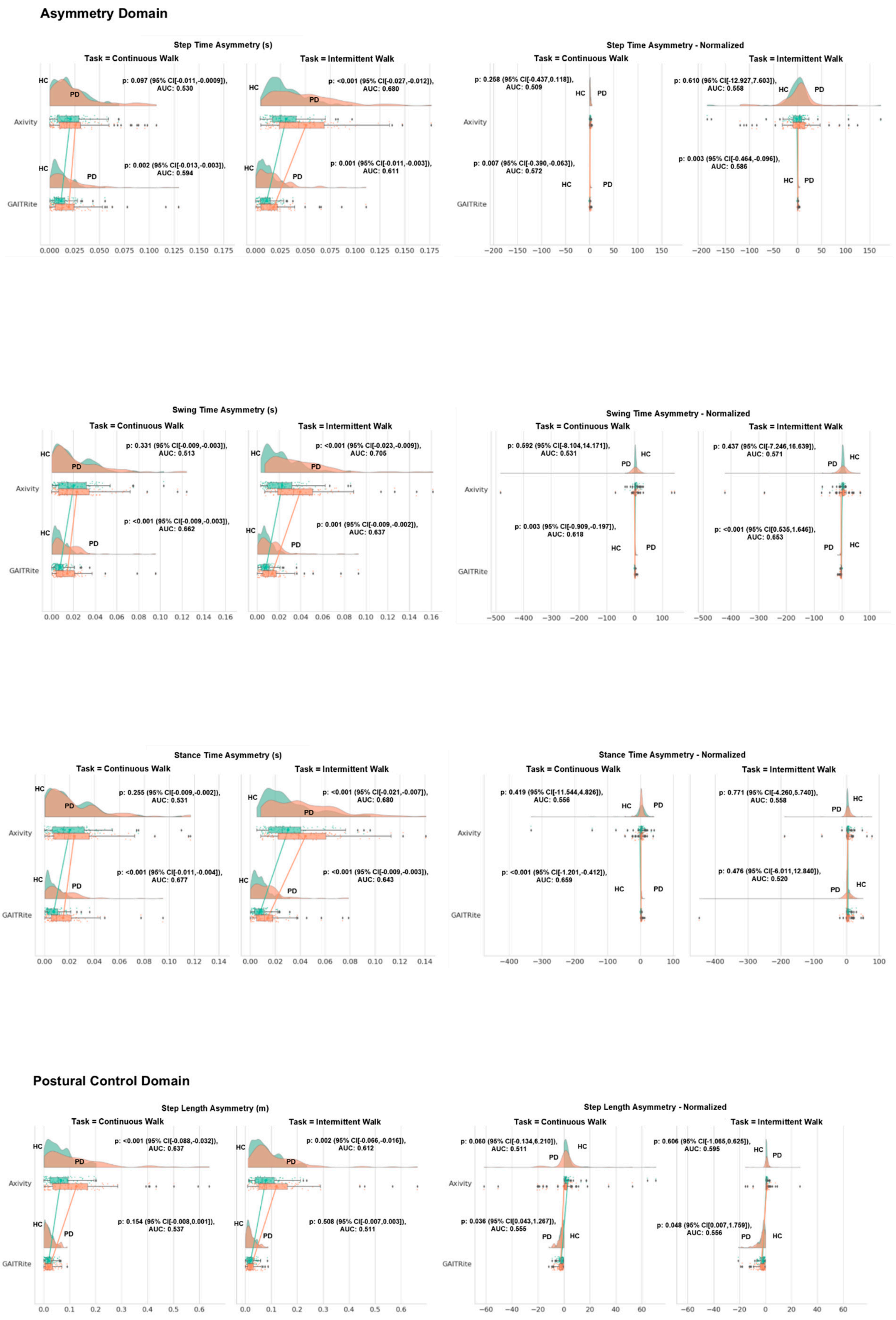
Asymmetry Domain
Step Time Asymmetry (s)
Task = Continuous Walk
Task = Intermittent Walk
p: 0.097 (95% CI[-0.011,-0.0009]), AUC: 0.530
p: <0.001 (95% CI[-0.027,-0.012]), AUC: 0.680
p: 0.002 (95% CI[-0.013,-0.003]), AUC: 0.594
p: 0.001 (95% CI[-0.011,-0.003]), AUC: 0.611
Step Time Asymmetry - Normalized
p: 0.258 (95% CI[-0.437,0.118]), AUC: 0.509
p: 0.610 (95% CI[-12.927,7.603]), AUC: 0.558
p: 0.007 (95% CI[-0.390,-0.063]), AUC: 0.572
p: 0.003 (95% CI[-0.464,-0.096]), AUC: 0.586
Swing Time Asymmetry (s)
p: 0.331 (95% CI[-0.009,-0.003]), AUC: 0.513
p: <0.001 (95% CI[-0.023,-0.009]), AUC: 0.705
p: <0.001 (95% CI[-0.009,-0.003]), AUC: 0.662
p: 0.001 (95% CI[-0.009,-0.002]), AUC: 0.637
Swing Time Asymmetry - Normalized
p: 0.592 (95% CI[-8.104,14.171]), AUC: 0.531
p: 0.437 (95% CI[-7.246,16.639]), AUC: 0.571
p: 0.003 (95% CI[-0.909,-0.197]), AUC: 0.618
p: <0.001 (95% CI[0.535,1.646]), AUC: 0.653
Stance Time Asymmetry (s)
p: 0.255 (95% CI[-0.009,-0.002]), AUC: 0.531
p: <0.001 (95% CI[-0.021,-0.007]), AUC: 0.680
p: <0.001 (95% CI[-0.011,-0.004]), AUC: 0.677
p: <0.001 (95% CI[-0.009,-0.003]), AUC: 0.643
Stance Time Asymmetry - Normalized
p: 0.419 (95% CI[-11.544,4.826]), AUC: 0.556
p: 0.771 (95% CI[-4.260,5.740]), AUC: 0.558
p: <0.001 (95% CI[-1.201,-0.412]), AUC: 0.659
p: 0.476 (95% CI[-6.011,12.840]), AUC: 0.520
Postural Control Domain
Step Length Asymmetry (m)
p: <0.001 (95% CI[-0.088,-0.032]), AUC: 0.637
p: 0.002 (95% CI[-0.066,-0.016]), AUC: 0.612
p: 0.154 (95% CI[-0.008,0.001]), AUC: 0.537
p: 0.508 (95% CI[-0.007,0.003]), AUC: 0.511
Step Length Asymmetry - Normalized
p: 0.060 (95% CI[-0.134,6.210]), AUC: 0.511
p: 0.606 (95% CI[-1.065,0.625]), AUC: 0.595
p: 0.036 (95% CI[0.043,1.267]), AUC: 0.555
p: 0.048 (95% CI[0.007,1.759]), AUC: 0.566
HC
PD
Axivity
GAITRite

Figure S2. Correlations among gait characteristics before and after normalization

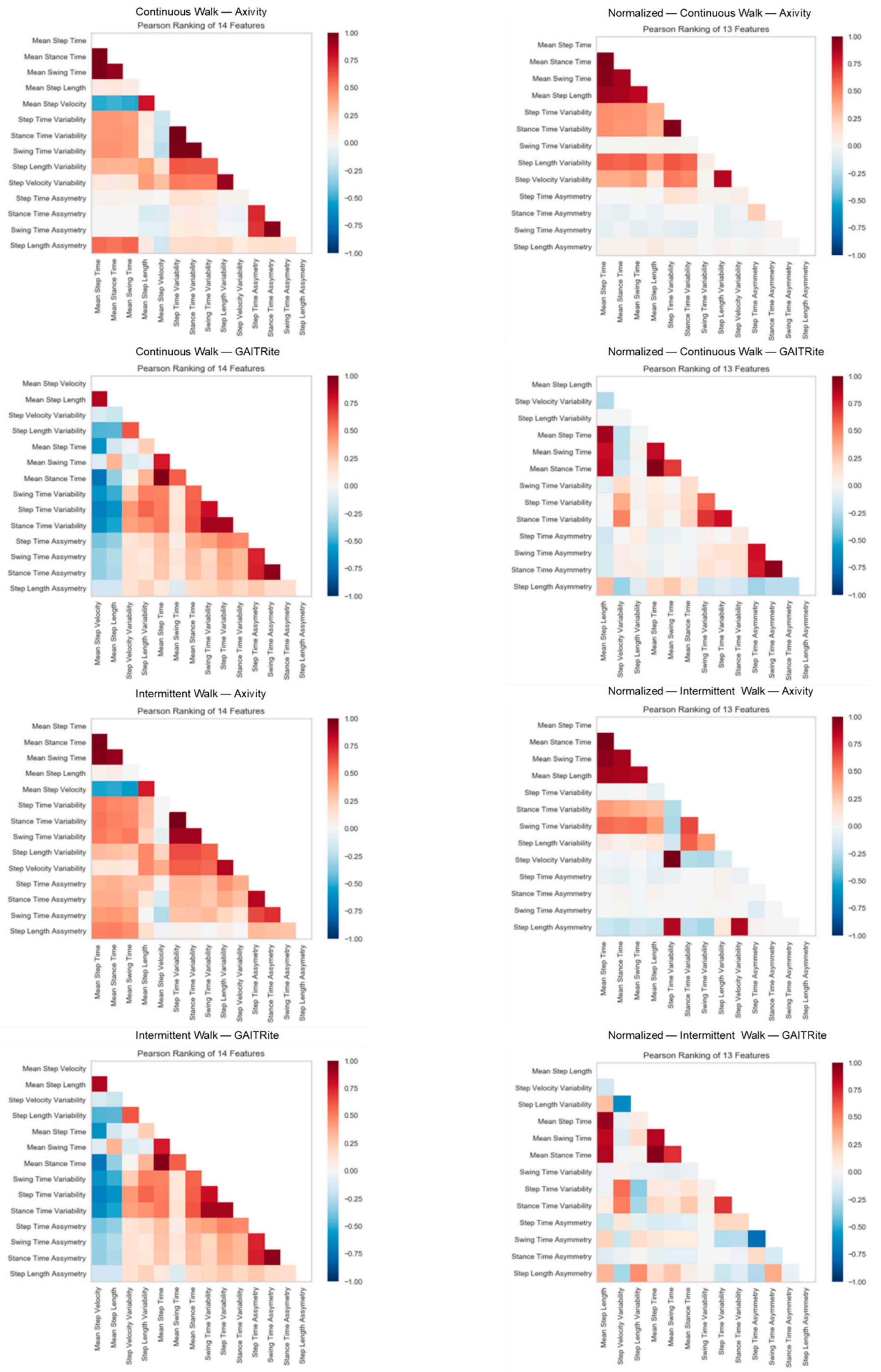

Figure S3. Contribution of the gait characteristics in the classification modelling in Support vector machine

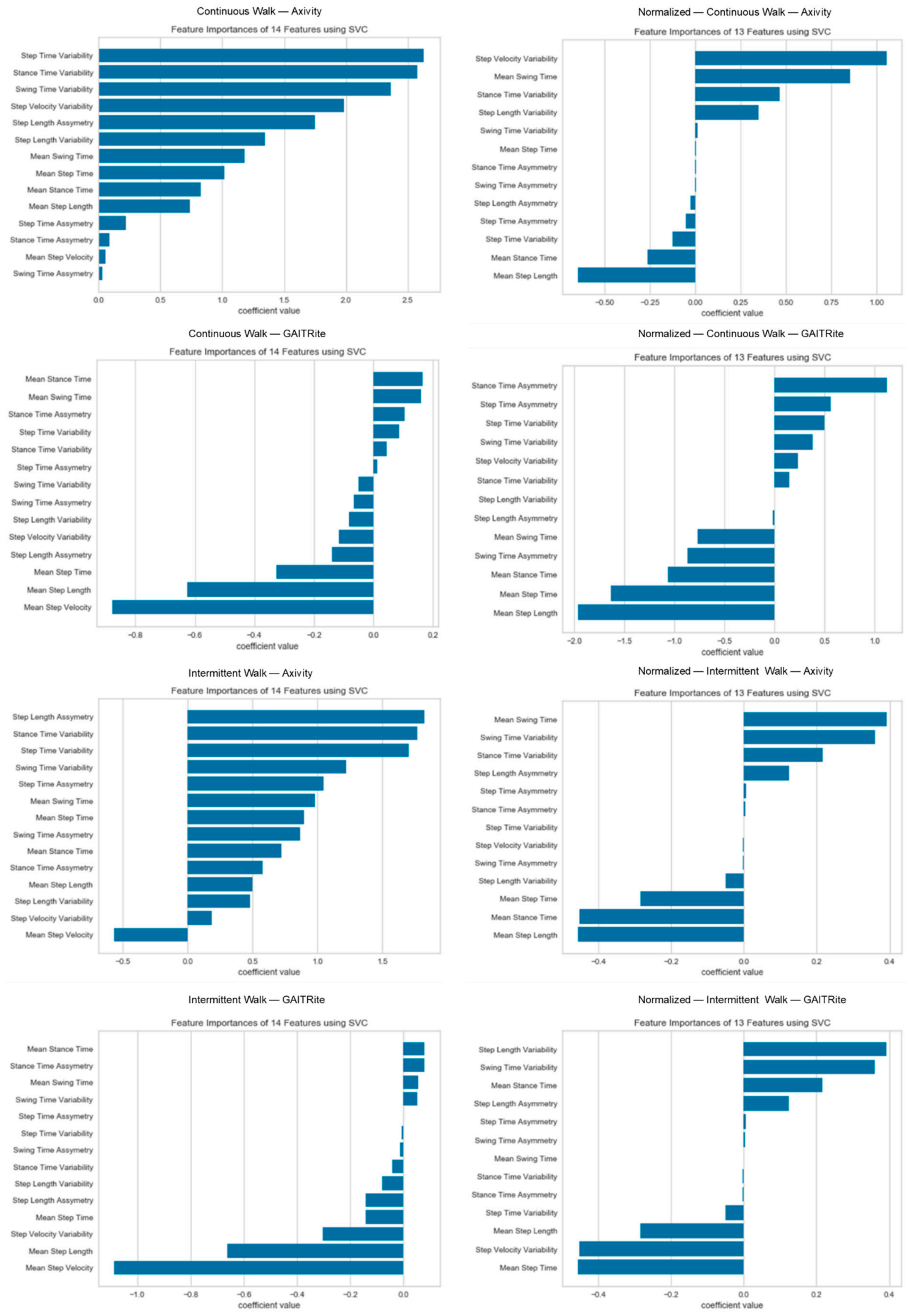

Continuous Walk — Axivity
Feature Importances of 14 Features using SVC
Step Time Variability
Stance Time Variability
Swing Time Variability
Step Velocity Variability
Step Length Assymetry
Step Length Variability
Mean Swing Time
Mean Step Time
Mean Stance Time
Mean Step Length
Step Time Assymetry
Stance Time Assymetry
Mean Step Velocity
Swing Time Assymetry
coefficient value
Normalized — Continuous Walk — Axivity
Feature Importances of 13 Features using SVC
Step Velocity Variability
Mean Swing Time
Stance Time Variability
Step Length Variability
Swing Time Variability
Mean Step Time
Stance Time Asymmetry
Swing Time Asymmetry
Step Length Asymmetry
Step Time Asymmetry
Step Time Variability
Mean Stance Time
Mean Step Length
coefficient value
Continuous Walk — GAITRite
Feature Importances of 14 Features using SVC
Mean Stance Time
Mean Swing Time
Stance Time Assymetry
Step Time Variability
Stance Time Variability
Step Time Assymetry
Swing Time Variability
Swing Time Assymetry
Step Length Variability
Step Velocity Variability
Step Length Assymetry
Mean Step Time
Mean Step Length
Mean Step Velocity
coefficient value
Normalized — Continuous Walk — GAITRite
Feature Importances of 13 Features using SVC
Stance Time Asymmetry
Step Time Asymmetry
Step Time Variability
Swing Time Variability
Step Velocity Variability
Stance Time Variability
Step Length Variability
Step Length Asymmetry
Mean Swing Time
Swing Time Asymmetry
Mean Stance Time
Mean Step Time
Mean Step Length
coefficient value
Intermittent Walk — Axivity
Feature Importances of 14 Features using SVC
Step Length Assymetry
Stance Time Variability
Step Time Variability
Swing Time Variability
Step Time Assymetry
Mean Swing Time
Mean Step Time
Swing Time Assymetry
Mean Stance Time
Stance Time Assymetry
Mean Step Length
Step Length Variability
Step Velocity Variability
Mean Step Velocity
coefficient value
Normalized — Intermittent Walk — Axivity
Feature Importances of 13 Features using SVC
Mean Swing Time
Swing Time Variability
Stance Time Variability
Step Length Asymmetry
Step Time Asymmetry
Stance Time Asymmetry
Step Time Variability
Step Velocity Variability
Swing Time Asymmetry
Step Length Variability
Mean Step Time
Mean Stance Time
Mean Step Length
coefficient value
Intermittent Walk — GAITRite
Feature Importances of 14 Features using SVC
Mean Stance Time
Stance Time Assymetry
Mean Swing Time
Swing Time Variability
Step Time Assymetry
Step Time Variability
Swing Time Assymetry
Stance Time Variability
Step Length Variability
Step Length Assymetry
Mean Step Time
Step Velocity Variability
Mean Step Length
Mean Step Velocity
coefficient value
Normalized — Intermittent Walk — GAITRite
Feature Importances of 13 Features using SVC
Step Length Variability
Swing Time Variability
Mean Stance Time
Step Length Asymmetry
Step Time Asymmetry
Swing Time Asymmetry
Mean Swing Time
Stance Time Variability
Stance Time Asymmetry
Step Time Variability
Mean Step Length
Step Velocity Variability
Mean Step Time
coefficient value

Figure S4. SVM performance before gait characteristics normalization

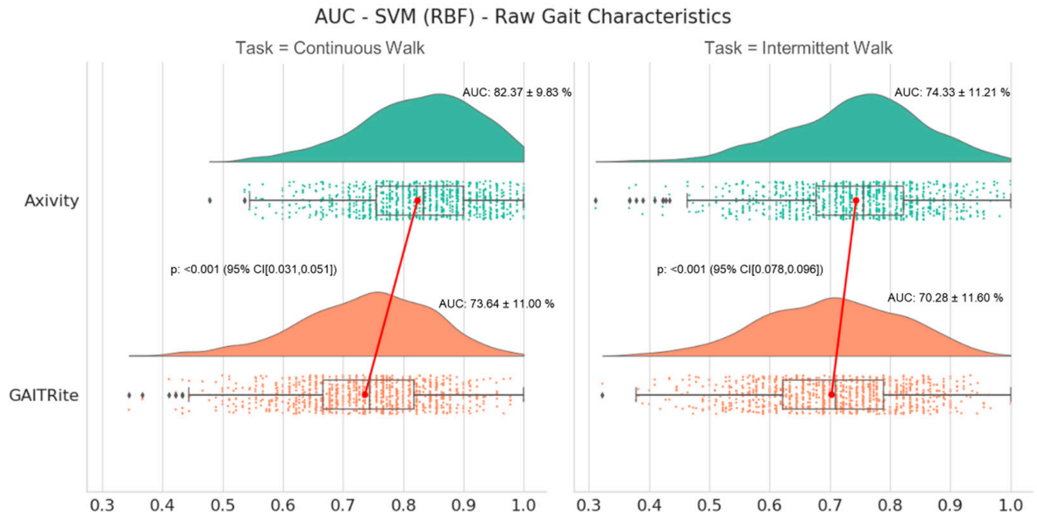

Supplement: Supplementary file 1 [file sensors-19-05363-s001.pdf]
